# Analyzing the Quality and Readability of Online Hyaluronic Acid Knee Injection Resources

**DOI:** 10.7759/cureus.43225

**Published:** 2023-08-09

**Authors:** Steven R Carlson, Chandler Sparks, Riya Savla, Ari Seidenstein, Gregg R Klein

**Affiliations:** 1 Orthopedic Surgery, Hackensack Meridian School of Medicine, Nutley, USA; 2 Orthopedic Surgery, Hackensack Meridian School of Medicine, Hackensack, USA; 3 Orthopedic Surgery, Hackensack University Medical Center, Hackensack, USA

**Keywords:** discern, osteoarthritis, knee injection, hyaluronic acid, viscosupplementation

## Abstract

Introduction: We analyzed the quality of information about Hyaluronic acid (HA) knee injections for osteoarthritis using DISCERN, a tool that grades the quality of websites. We also analyzed readability with Flesch-Kincaid grade reading levels (FKGRL).

Methods: Lists of the top ten included sites from Google searches about HA injections were evaluated using DISCERN to determine their quality. Additional variables collected were site category, Health on Net (HON) certification, search result rank, and FKGRL. DISCERN scores were compared and grouped by these variables.

Results: Most sites were measured as fair in quality. Greater DISCERN scores were produced from searches using general terminology, sites with HON labels, and academic journal publications.

Conclusion: This study indicates information quality for HA injections online is fair. The data also indicates that patients can best educate themselves using HON labels, general search terms, and information from academic journals when possible.

## Introduction

Osteoarthritis is a chronic disease affecting several joints in the body, very frequently the shoulder, hip, or knee. Healthy articular surfaces of these joints become worn away as articular cartilage production cannot match the degradation of collagen and proteoglycan components by digestive enzymes [1]. In some management instances, patients opt for a therapy that addresses this pathophysiology, such as viscoelastic supplements (viscosupplementation) in the form of hyaluronic acid (HA) injection. HA is a naturally occurring molecule that provides lubrication in bodily tissues such as the synovial fluid of joints [2] and also has been shown to restore levels of proteoglycan synthesis for osteoarthritis management [3].

Though HA has been injected intraarticularly in the knee for over three decades, there remains significant controversy in its usage. While the American Academy of Orthopedic Surgeons has consistently provided recommendations against its usage, one systematic review reported that 74% of national and professional societies recommended the use of HA knee injections [4]. In light of this, these injections are given to one in every seven patients with knee osteoarthritis [5], and insurance expenditures on these products have been shown to increase over recent years [6] steadily. As a result, the marketplace for HA injection products has become quite diverse in terms of branding or regimen. The quantity of products combined with recommendations lacking consistency creates an overwhelming experience for patients learning about this therapy.

Patients regularly turn to internet search engines such as Google [7] for research in the information era. Though a Google search provides no shortage of websites, it remains a challenge to distinguish between low- or high-quality sources, particularly regarding health information. To address this, a web tool called DISCERN was developed to systematically evaluate the quality of information about diseases and their management using a series of 16 questions graded from one to five. These questions evaluate for bias, reliability, authorship, and relevance. Since DISCERN and similar tools can be quite extensive, consumer-friendly tools such as the Health on Net Foundation Code of Conduct (HONcode) have also been created to systematically analyze and supply a seal of approval to high-quality websites. In several studies evaluating the quality of information pertinent to osteoarthritis [8,9] or other disease processes and their treatment options, DISCERN and HONcode have been used.

In this study, we used DISCERN and HONCode labels along with each site's Flesch-Kincaid grade reading level to evaluate the quality and readability of information available to patients regarding HA knee injections. In doing so, we compared these scores across various variables, including the search keywords and the category or order of appearance of results. Due to findings from previous studies, we hypothesized that the websites' quality and readability would score poorly across several variables.

## Materials and methods

Search strategy

This study used Google Chrome incognito mode to obtain a list of websites to analyze. Incognito mode prevents previously tracked history information from biasing the search process. Additional Chrome preferences, such as location tracking and history recording, were also disabled. Importantly, no means were available to disable Google's ability to identify locations acquired by IP address. On March 27, 2023, seven Google searches were performed using either generic terminology or the brand names of specific HA injections as keywords. Of the three generic searches, the terms "Viscosupplementation knee injection", "Gel knee injection", and "Hyaluronic acid knee injection" were used. In the four brand-specific searches, the terms "Orthovisc knee injection", "Durolane knee injection", "Synvisc one knee injection", and "Euflexxa knee injection" were used. These four brands were selected as keywords in the search process due to their popularity as measured by the total number of reviews found on Drugs.com (https://www.drugs.com/drug-class/viscosupplementation-agents.html).

 List creation

Using the following exclusion criteria to generate the list, ten websites in order from top to bottom of the search results were compiled for each search. Sites were excluded from each recorded list if they were irrelevant to the topic, directed to another tab of a site already recorded, or if they directed to a PDF document, video, or PowerPoint. Sites that specifically indicated purpose for healthcare professionals and sites that indicated sponsorship were also excluded. Once ten search results were included in the list for each search, no further search results were assessed for eligibility. The URLs of included sites for each keyword search were extracted into an Excel spreadsheet and assembled into a scrambled list for review.

During list creation, the site category was recorded as an academic institution, academic journal, commercial, government/patient advocacy, non-academic medical practice, or other based on criteria similar to those observed in an article by Sullivan et al. [9]. Additional variables recorded in the search process were the presence of HONCode approval and Flesch-Kincaid grade reading levels. The HONcode Toolbar Chrome extension was used to identify approved sites, while the CraftyLevel Chrome extension was used to obtain Flesch-Kincaid ratings.

DISCERN scoring

Two independent reviewers evaluated the quality of each of the 60 included sites from the scrambled list using the DISCERN tool. Scores for Questions 1-15 were summed to assign a total score for each rater, excluding Question 16, which asks reviewers to give a composite score of the entire website as was done in a previous study [10]. Mean total DISCERN scores were then determined for each site using the score from each rater. According to the previous study, these total scores were categorized as follows: 0-27 was very poor, 28-38 was poor, 39-50 was fair, 51-62 was good, and above 62 was excellent.

Statistical analysis

All statistical analyses were performed in GraphPad Prism version 9.5.1 (528), January 24, 2023. Interrater reliability of DISCERN scores was measured using Spearman's rho nonparametric correlation coefficient with 95% confidence intervals. Descriptive statistical analysis calculated means +/- standard deviation of DISCERN and Flesch-Kincaid ratings. The Mann-Whitney test was used to compare mean DISCERN scores. DISCERN scores of variables with multiple categories were compared using Kruskal-Wallis and Dunn's multiple comparisons tests. Additional continuous variables were analyzed using Spearman's correlation coefficients. The statistical significance of results from these tests was set a priori as those with P-values ≤ 0.05.

## Results

Search results and quality/readability scores

**Figure 1:**
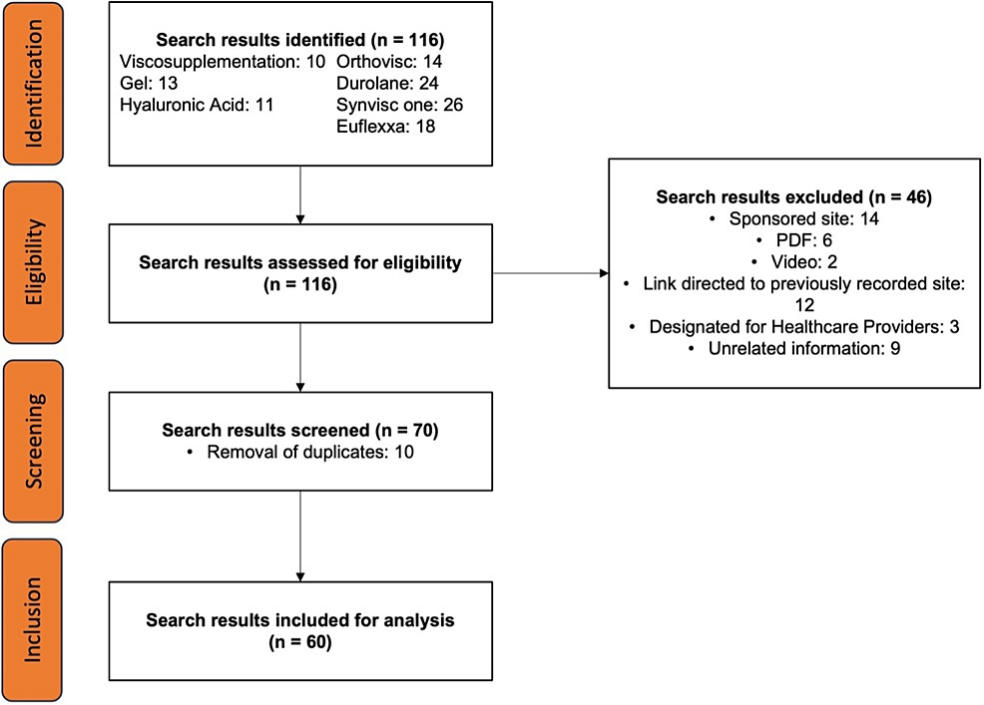
Flow diagram of search strategy using 7 unique searches and exclusion criteria to narrow Google search results.

Our search yielded 116 sites, of which 46 were excluded due to meeting the above criteria (Fig 1). On average, general search terms required the exclusion of 1.33 websites per search, while brand name search terms required the exclusion of 10.5 websites per search. Once a list of the top ten included results for each search was obtained for the seven searches, 70 sites remained. Of the 70 websites included, ten were duplicates or triplicates found in other searches, leaving 60 unique sites to analyze. Investigating the connection between quality and readability, the correlation analysis between the Flesch-Kincaid readability score and DISCERN quality revealed no significant relationship (Spearman's ρ = 0.1896, 95% CI: -0.08 to 0.43). This testing was also used to understand if sites that were transparent about the lack of consistent recommendations for HA supplementation usage had greater DISCERN scores. The correlation analysis between DISCERN scores for question eight and total DISCERN scores showed a strong relationship (Spearman's ρ = 0.7420,95% CI: 0.60 to 0.84), indicating a relationship between transparency and overall quality.

**Figure 2:**
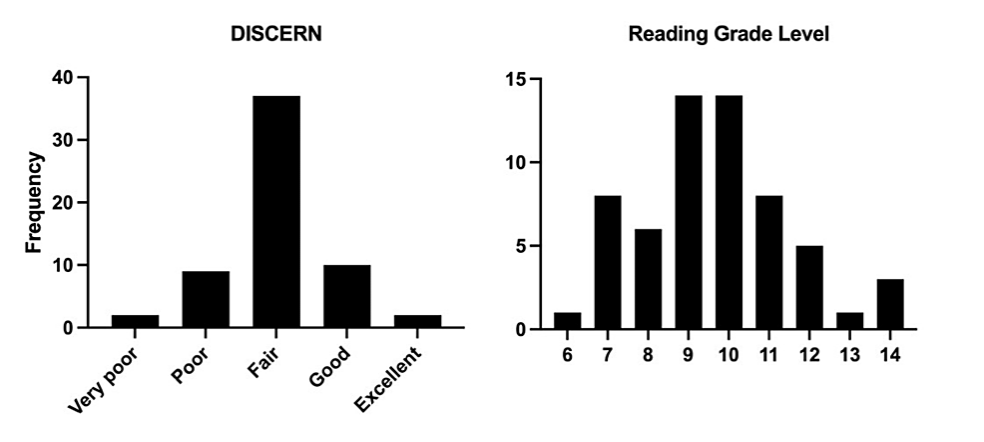
Histograms showing frequencies of included sites (n=60) within each DISCERN quality category and Flesch-Kincaid grade reading level.

After scoring each of the 60 unique sites by raters, correlation analysis concerning the DISCERN scores from each of the two independent raters found strong agreement (Spearman's ρ = 0.57, 95% CI: 0.36 to 0.72). Among all sites reviewed, the mean DISCERN score was 45.43 +/- 8.53 indicating overall fair quality. Moreover, 37 (61.67%) of all articles reviewed were designated as fair in quality (Fig 2). Of the 60 sites assessed, only 12 (20%) were good quality or better, and 11 (18.33%) were poor quality or worse. As for the readability of these 60 sites, nearly half (46.67%) had Flesch-Kincaid grade reading scores between grades nine and ten (Fig 2). The mean grade reading level was 9.63 +/- 1.88.

Characterization of factors that affect the quality of sites

**Figure 3:**
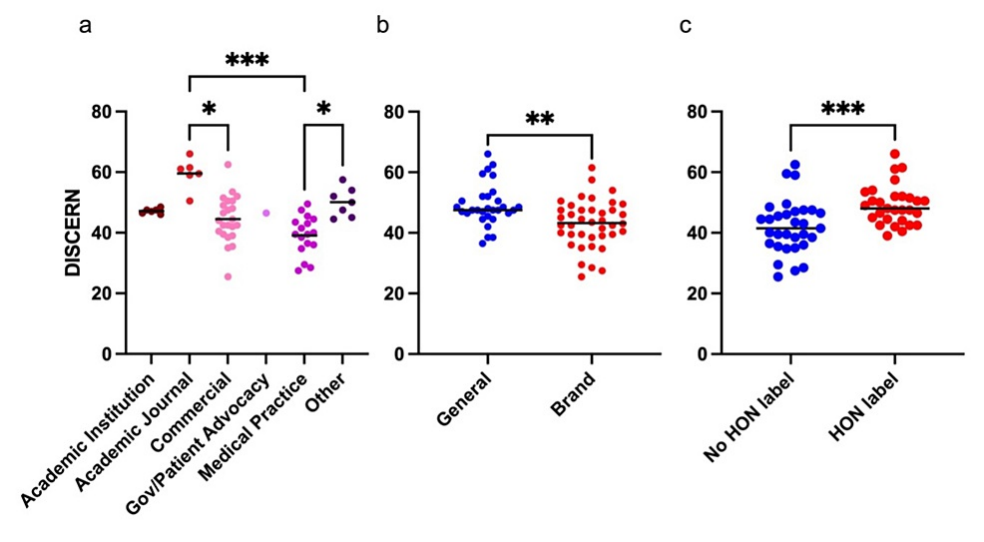
DISCERN scores represented as a function of website category (a), keyword search category (b), and presence or absence of HON certification label (c). Each subgroup of DISCERN scores is presented with a mean. Pairwise comparisons from Kruskal-Wallis with Dunn’s multiple comparisons testing (a) and Mann-Whitney testing (b and c) are presented for statistically significant results. *P ≤ 0.05; **P ≤ 0.01; ***P ≤ 0.001. HON = Health on Net

After dividing websites into five categories, six sites were determined to be from academic institutions, six were from academic journals, 23 were from commercial websites, one was from a patient advocacy group, and seven were from other sites. Four sites within the other category were articles on the same social media site (www.verywellhealth.com). The highest mean DISCERN score was 59.58 +/- 5.09 from academic journals, and the lowest was 39.13 +/- 6.45 from medical practices. Academic journals were the only category that met the criteria to be labeled as good quality, while the rest were of fair quality. Excluding academic journal articles from the entire sample, the overall mean DISCERN score was reduced from 45.34 +/- 8.53 to 43.83 +/- 7.33. ANOVA comparing the DISCERN scores of these categories of websites showed three significant differences (Fig 3a). Academic journals had higher DISCERN scores than commercial sites (p=0.12) and medical practices (p<0.001). Also, sites from the other category had greater DISCERN scores than those from medical practices.

**Figure 4:**
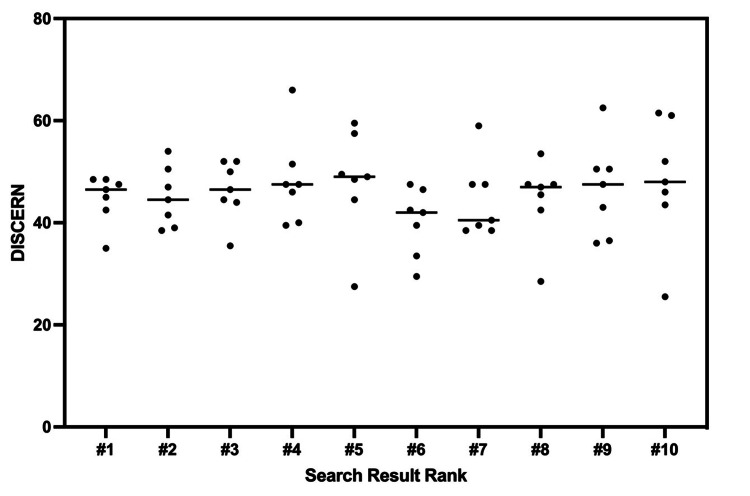
DISCERN scores from websites grouped and represented as a function of their order of appearance on Google search. Each search result rank group is presented with a mean.

To determine the impact that other variables had on quality, DISCERN scores were assessed as a function of these variables. Both general and brand name search terms led to fair scores in the aggregate; however, general search terms did yield greater quality results (p=0.0016) (Fig 3b). Specifically, general search terms yielded a mean DISCERN score of 49.15 +/- 6.90, while brand names yielded a mean score of 43.04 +/- 7.84. DISCERN scores of sites with HONcode certification labels were also found to be fair in quality, with a mean score of 49.16 +/- 6.46 which was greater than sites with no label that had a mean of 41.94 +/- 8.84 (p=0.0002) (Fig 3c). Last, the relationship between search result appearance on Google and DISCERN score was compared. Using ANOVA to compare the mean discern scores, it was discovered that there were no significant differences between any of the groups of search results from one to ten (p=0.566) (Fig 4).

Investigating the connection between quality and readability, the correlation analysis between the Flesch-Kincaid readability score and DISCERN quality revealed no significant relationship (Spearman's ρ = 0.1896, 95% CI: -0.08 to 0.43). This testing was also used to understand if sites that were transparent about the lack of consistent recommendations for HA supplementation usage had greater DISCERN scores. The correlation analysis between DISCERN scores for question eight and total DISCERN scores showed a very strong relationship (Spearman's ρ = 0.7420, 95% CI: 0.60 to 0.84), indicating a relationship between transparency and overall quality.

## Discussion

This study found that the quality of information returned from a Google search for HA knee injections is of fair quality. While some websites were of good or excellent quality (20%), most were of fair quality (~62%), and some were of poor or very poor quality (~18%). Factors predictive of DISCERN score include website source, search term, and HONcode certification label. Regarding website sources, academic journals were found to be of high quality, whereas commercial and medical practice sites were of lower quality. This finding is unsurprising given that academic journals aim to disseminate high-quality, trustworthy information, and a thorough review of articles in these journals is common practice. This, however, is not true of commercial sites/medical practices, which often have other goals such as advertisement of their products. This being the case, our results contrast similar studies investigating online information about knee osteoarthritis, which found commercial and academic sites to have higher quality information than non-academic medical practice and government/patient advocacy websites [9,11,12].

Regarding search terms, general terms provided higher quality websites than brand name search terms. General search terms also yielded fewer sites that met exclusion criteria than brand name search terms, indicating lesser heterogeneity of websites. Though in this study, several sites were excluded, such as those unrelated or from sponsored sources, the reality is that patients would be very likely to encounter these sources, especially if they were found in the first ten results. Fortunately, we could show that the first ten eligible search results were not significantly different in terms of quality.

The finding that HONcode certification labels indicate high-quality websites was predictable, given assessment and approval by an outside organization. Checking for this label is a very simple method for anyone searching Google to ensure that the websites contain high-quality information. In addition, those searching for HA injection information would also benefit from understanding that this treatment option remains questionable. The very strong relationship between the DISCERN question 8 score, which indicates transparency about the treatment option, and the overall DISCERN score demonstrates that articles acknowledging viscosupplementation's questionable utility should receive a higher value.

Even if a site's information is deemed high quality, patients with low health literacy still may be unable to utilize this information. Journal articles, for instance, found to be of the highest quality in this study, remain concerning regarding readability. Unfortunately, as low readability affects many patients' understanding of this information, they are often challenged to make decisions about their treatment and develop worse health outcomes [13,14]. Though we found no correlation between Flesch-Kincaid grade reading level and site quality, all sites' mean Flesch-Kincaid grade reading level was between 9th and 10th grade. This score is higher than the average U.S. citizen's 8th-grade reading level [15] and falls above the NIH recommendation for patient educational information to be between the 7th to 8th-grade reading level [16]. In orthopedics, lack of readability is common [17], and our results show improvement has yet to occur.

## Conclusions

This study characterized the quality and readability of online information about HA knee injections found via a Google search while identifying factors influencing quality. Strengths of this study include the strong degree of interrater agreement using the DISCERN scoring system and the analysis of several key search variables that can help patients locate the best information regarding this controversial treatment. There are also limitations to address for this study. First, including academic journal articles may have led to overestimating the aggregate quality scores. Excluding those articles from the website sample, the mean DISCERN score remained fair, indicating that this overestimate was relatively minor. Also, the completion of quality scoring by medical students may have influenced how each site was rated due to their medical education. Altogether, patients should be advised usage of general search terms and the HONcode certification label as an indicator of quality when researching HA injections. Patients can best educate themselves using academic journals when readable, or if these are unreadable, by avoiding commercial or medical practice websites.
